# Low-dose metronomic chemotherapy as an efficient treatment option in metastatic breast cancer—results of an exploratory case–control study

**DOI:** 10.1007/s10549-020-05711-5

**Published:** 2020-06-03

**Authors:** S. Krajnak, C. Schnatz, K. Almstedt, W. Brenner, F. Haertner, A.-S. Heimes, A. Lebrecht, G.-M. Makris, R. Schwab, A. Hasenburg, M. Schmidt, M. J. Battista

**Affiliations:** 1 Department of Gynecology and Obstetrics, University Medical Center Mainz, Langenbeckstrasse 1, 55131 Mainz, Germany; 2 Institute of Medical Biometry, Epidemiology and Informatics (IMBEI), University Medical Center Mainz, Mainz, Germany

## Abstract

**Purpose:**

There is growing interest in low-dose metronomic chemotherapy (LDMC) in metastatic breast cancer (MBC). In this retrospective case–control analysis, we compared the efficacy of LDMC and conventional chemotherapy (CCT) in MBC.

**Methods:**

Each LDMC patient receiving oral cyclophosphamide (CTX) (50 mg daily) and methotrexate (MTX) (2.5 mg every other day) was matched with two controls who received CCT. Age, number of chemotherapy lines and metastatic sites as well as hormone receptor (HR) status were considered as matching criteria. Primary endpoint was disease control rate longer than 24 weeks (DCR). Secondary endpoints were progression-free survival (PFS), duration of response (DoR) and subgroup analyses using the matching criteria.

**Results:**

40 cases and 80 controls entered the study. 30.0% patients with LDMC and 22.5% patients with CCT showed DCR (*p* = 0.380). The median PFS was 12.0 weeks in both groups (*p* = 0.218) and the median DoR was 31.0 vs. 20.5 weeks (*p* = 0.383), respectively. Among younger patients, DCR was 40.0% in LDMC vs. 25.0% in the CCT group (*p* = 0.249). DCR was achieved in 33.3% vs. 26.2% non-heavily pretreated patients (*p* = 0.568) and in 36.0% vs. 18.0% patients without multiple metastases (*p* = 0.096), respectively. In the HR-positive group, 30.0% LDMC vs. 28.3% CCT patients showed DCR (*p* = 1.000). Among triple-negative patients, DCR was achieved in 30.0% LDMC and 5.0% CCT patients (*p* = 0.095).

**Conclusions:**

We demonstrated a similar efficacy of LDMC compared to CCT in the treatment of MBC. Thus, LDMC may be a valuable treatment option in selected MBC patients.

## Introduction

Metastatic breast cancer (MBC) is an incurable but treatable disease. Thus, it is crucial to achieve disease control with preservation of quality of life (QoL) [1]. In the last decades low-dose metronomic chemotherapy (LDMC) gained increasing popularity [2, 3]. LDMC is defined as a continuous administration of cytotoxic drugs at low doses, distinctly lower than the maximum tolerable dose (MTD) of conventional chemotherapy (CCT) [4]. Consequently, compared to MTD the lower doses of chemotherapeutic drugs may induce less adverse events like myelosuppression, mucositis or hair loss [5–7]. It is assumed that LDMC is not simply a different way of administering chemotherapy but a truly new treatment option [3, 8, 9]. This alternative strategy has been used especially in elderly patients, not eligible for a CCT [8]. The orally available and well-established cytostatic agents like cyclophosphamide (CTX), methotrexate (MTX), vinorelbine (VRL) and capecitabine (CAPE) are suited for metronomic chemotherapy. The best experience about LDMC arises from phase II studies, however phase III studies are still lacking. Furthermore, to the best of our knowledge, there is insufficient experience regarding the efficacy of metronomic chemotherapy, compared to CCT in MBC.

In this retrospective case–control study, the efficacy of metronomic administered CTX/MTX and CCT was compared in matched pairs and subgroup analyses were performed to define patients in which LDMC might be a more effective treatment option.

## Methods

MBC patients receiving LDMC with oral CTX (50 mg daily) and MTX (2.5 mg every other day) at the Department of Gynecology and Obstetrics of the University Medical Center Mainz, Germany between 2009 and 2018 were selected for this retrospective analysis as previously described [10]. Each LDMC patient was matched with two patients, who received CCT, if matching criteria (measurable metastatic disease, age at start of therapy, number of chemotherapy lines and different metastatic sites as well as hormone receptor (HR) status) were met. No antiemetic treatment was routinely given to patients in the LDMC group. In the CCT group only patients without therapy termination due to toxicity were included. No concomitant treatment like radiotherapy, endocrine or targeted therapy was allowed. HER2-positive patients and patients with presence of additional cancer were excluded.

Primary endpoint was disease control rate longer than 24 weeks (DCR). DCR included stable disease (SD), partial response (PR) and complete response (CR). Secondary endpoints were progression-free survival (PFS), duration of response (DoR), as well as DCR and PFS in subgroups. The DoR was defined as the time from documentation of tumor response to progression disease (PD) or death. The therapy efficacy was assessed using the standard clinical and imaging methods. For subgroup analyses we used the a priori determined matching criteria to obtain comparable populations of same size. Thereby, we stratified the patients by age at start of LDMC/CCT (younger: ≤ median age vs. elderly: > median age), the number of chemotherapy lines (non-heavily pretreated: ≤ 2 chemotherapy lines vs. heavily pretreated: > 2 chemotherapy lines), number of different metastatic sites (no multiple metastases: ≤ 2 different metastatic sites vs. multiple metastases: > 2 different metastatic sites) and by HR status (HR-positive: oestrogen/progesterone positive and HER2-negative vs. triple-negative). SPSS (statistical software system, version 23.0. IBM Corp., Armonk, NY, U.S.) was used for statistical analyses. Patient characteristics and therapy response (DCR and therapy response in subgroups) were analysed by applying a Fisher’s Exact test. For PFS and DoR analysis Kaplan–Meier estimator was used. The Log-rank test was used for the comparisons of survival curves between LDMC and CCT group. A Cox regression model was used to estimate the hazard ratio (HR) and 95% confidence interval (CI) in the analysis of PFS and DoR. All tests were two-sided and p < 0.05 was considered as statistically significant. Written informed consent was obtained from all patients included in the study.

## Results

### Patient characteristics

In total, 120 patients (40 cases and 80 controls) entered the study. Patient characteristics are shown in Table 1. The median age at first diagnosis (FD) of MBC was 59 (33–82) years in the LDMC group and 59 (28–81) years in the CCT group (*p* = 0.544). The median age at start of therapy was 63 (range 35–83) years and 61 (range 30–81) years (*p* = 0.230), respectively. In the HR-positive cohort, 93.3% LDMC patients and 71.7% CCT patients had at least one endocrine therapy in MBC prior to LDMC or CCT treatment (*p* = 0.026). 47.5% vs. 51.3% patients received adjuvant or neoadjuvant chemotherapy at FD of breast cancer (BC) (*p* = 0.847). After FD of MBC, 52.5% patients received less than 2 chemotherapy lines before LDMC/CCT (*p* = 1.000) (Table 1). 62.5% patients had up to 2 different metastatic lesions (*p* = 1.000). In the LDMC group as well as in the CCT group 25.0% patients showed triple-negative disease (*p* = 1.000). As demonstrated in Table 1, all matching criteria were met. The most frequent site of metastases in the LDMC group as well as in the CCT group were bone (62.5% vs. 70.0%, *p* = 0.417), liver (52.5% vs. 52.5%, *p* = 1.000) and lung (40% vs. 40%, *p* = 1.000), respectively. There were no significant differences regarding number and localization of metastatic lesions between the two groups (Table 2). In the CCT group, the most frequently used chemotherapy regimen was capecitabine (27.5%) and pegylated liposomal doxorubicin (26.3%). Taxanes were administered in 17.5% patients and eribulin in 12.5% patients (Table 3).

**Table 1:** Patient characteristics

	LDMC		CCT		*p*
	*n* = 40		*n* = 80		
Median age at start of therapy (range) (years)	63 (35–83)		61 (30–81)		0.230
Median age at FD MBC (range) (years)	59 (33–82)		59 (28–81)		0.544
Median age at FD BC (range) (years)	51 (29–80)		52 (26–79)		0.506
Age at start of therapy					
Younger	20 (50.0%)		40 (50.0%)		1.000
Elderly	20 (50.0%)		40 (50.0%)		
Chemotherapy line					
Non-heavily pretreated	21 (52.5%)		42 (52.5%)		1.000
Heavily pretreated	19 (47.5%)		38 (47.5%)		
Metastatic sites					
No multiple metastases	25 (62.5%)		50 (62.5%)		1.000
Multiple metastases	15 (37.5%)		30 (37.5%)		
HR status					
HR-positive	30 (75.0%)		60 (75.0%)		1.000
Triple-negative	10 (25.0%)		20 (25.0%)		

*LDMC* low-dose metronomic chemotherapy, *CCT* conventional chemotherapy, *FD* first diagnosis, *MBC* metastatic breast cancer, *BC* breast cancer, *HR* hormone receptor

**Table 2:** Localization of metastatic lesions

		LDMC		CCT	p
Metastatic sites		No. of patients (%)			
Bone		25 (62.5%)		56 (70.0%)	0.417
Liver		21 (52.5%)		42 (52.5%)	1.000
Lung		16 (40.0%)		32 (40.0%)	1.000
Pleura		7 (17.5%)		10 (12.5%)	0.579
Peritoneum		2 (5.0%)		9 (11.3%)	0.333
Lymph		12 (30.0%)		23 (28.8%)	1.000
Cerebrum		1 (2.5%)		8 (10.0%)	0.269
Soft tissue (thoracic wall, cutis)		5 (12.5%)		10 (12.5%)	1.000

**Table 3:** Chemotherapeutic substances in the conventional chemotherapy group

Chemotherapy	No. of patients (%)
Capecitabine	22 (27.5%)
Pegylated liposomal doxorubicin	21 (26.3%)
Taxane (paclitaxel/nab-paclitaxel/docetaxel)	14 (17.5%) (4 (5.0%)/ 8 (10.0%)/ 2 (2.5%))
Eribulin	10 (12.5%)
Carboplatin + gemcitabine	5 (6.3%)
Vinorelbine	4 (5.0%)
Other (doxorubicin/carboplatin/fluorouracil/capecitabine + vinorelbine)	4 (5.0%) (each 1 (1.3%))

### Therapy response

**Fig. 1:**
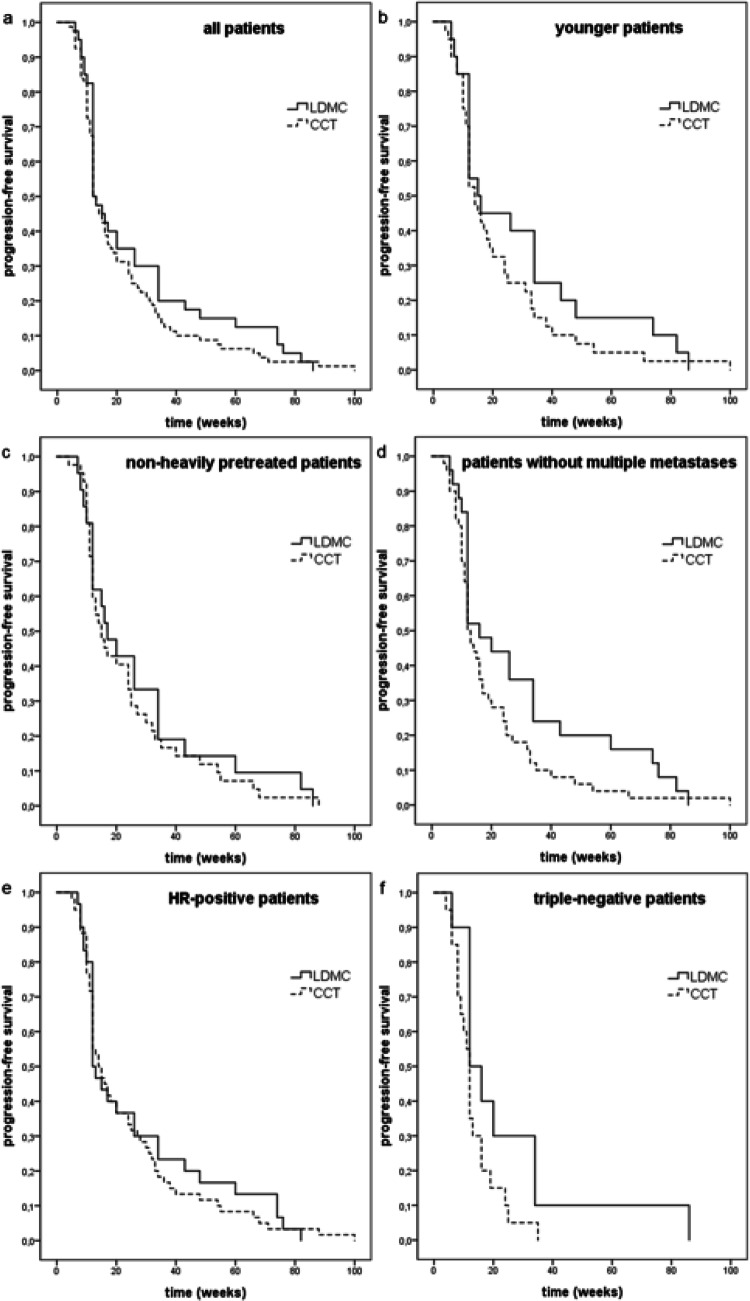
Kaplan–Meier analysis of progression-free survival. **a** all patients: median PFS in LDMC and CCT: 12.0 weeks vs. 12.0 weeks, Log-rank: *p* = 0.218. **b** younger patients: median PFS in LDMC and CCT: 15.0 weeks vs. 14.0 weeks, Log-rank: *p* = 0.212. **c** non-heavily pretreated patients: median PFS in LDMC and CCT: 17.0 weeks vs. 15.0 weeks, Log-rank: *p* = 0.531. **d** patients without multiple metastases: median PFS in LDMC and CCT: 16.0 weeks vs. 12.0 weeks, Log-rank: *p* = 0.064. **e** HR-positive patients: median PFS in LDMC and CCT: 12.0 weeks vs. 14.0 weeks, Log-rank: *p* = 0.570. **f** triple-negative patients: median PFS in LDMC and CCT: 12.0 weeks vs. 12.0 weeks, Log-rank: *p* = 0.081. *LDMC* low-dose metronomic chemotherapy), *CCT* conventional chemotherapy

DCR was achieved in 30.0% LDMC patients and in 22.5% CCT patients (*p* = 0.380) (Table 2). 12.5%, 15.0%, 2.5% LDMC patients vs. 18.8%, 3.8%, 0.0% CCT patients showed SD, PR, CR, respectively (Table 4). The median PFS was 12.0 weeks (95% CI 9.9–14.1) in the LDMC group, as compared with 12.0 weeks (95% CI 10.5–13.5) in the CCT group, HR for progression or death was 0.796; 95% CI 0.541–1.170; *p* = 0.245 (Fig. 1). The median DoR was 31.0 weeks in the LDMC group and 20.5 weeks in the CCT group (*p* = 0.383) (Table 4), HR for progression or death was 0.749; 95% CI 0.385–1.459; *p* = 0.396. Therapy response was detected in 37.5% LDMC patients and in 30.0% CCT patients (*p* = 0.417) (Table 4).

**Table 4:** Therapy response

		LDMC	CCT	*p*
		*n* = 40	*n* = 80	
DCR (*n* (%))		12 (30.0%)	18 (22.5%)	0.380
Therapy response after 24 weeks	PD	28 (70.0%)	62 (77.5%)	
	SD	5 (12.5%)	15 (18.8%)	
	PR	6 (15.0%)	3 (3.8%)	
	CR	1 (2.5%)	0 (0.0%)	
Median PFS (range) (weeks)		12.0 (6–86)	12.0 (4–100)	0.218
Median duration of response (range) (weeks)		31.0 (12–74)	20.5 (12–88)	0.383
Therapy response (n (%))		15 (37.5%)	24 (30.0%)	0.417

*DCR* Disease Control Rate, *PFS* progression-free survival, *PD* progression disease, *SD* stable disease, *PR* Partial response, *CR* Complete response

### Therapy response in subgroups

**Fig. 2:**
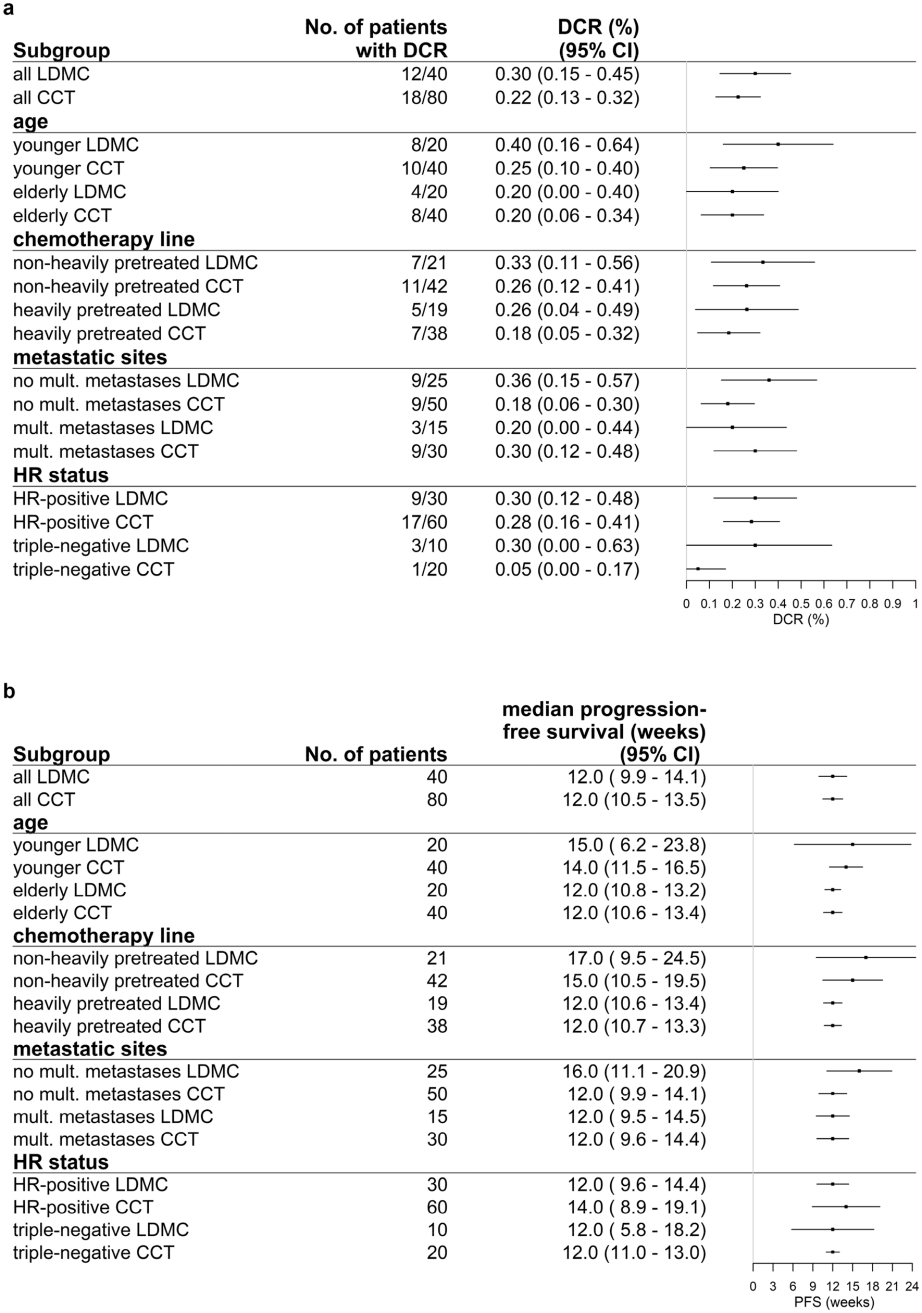

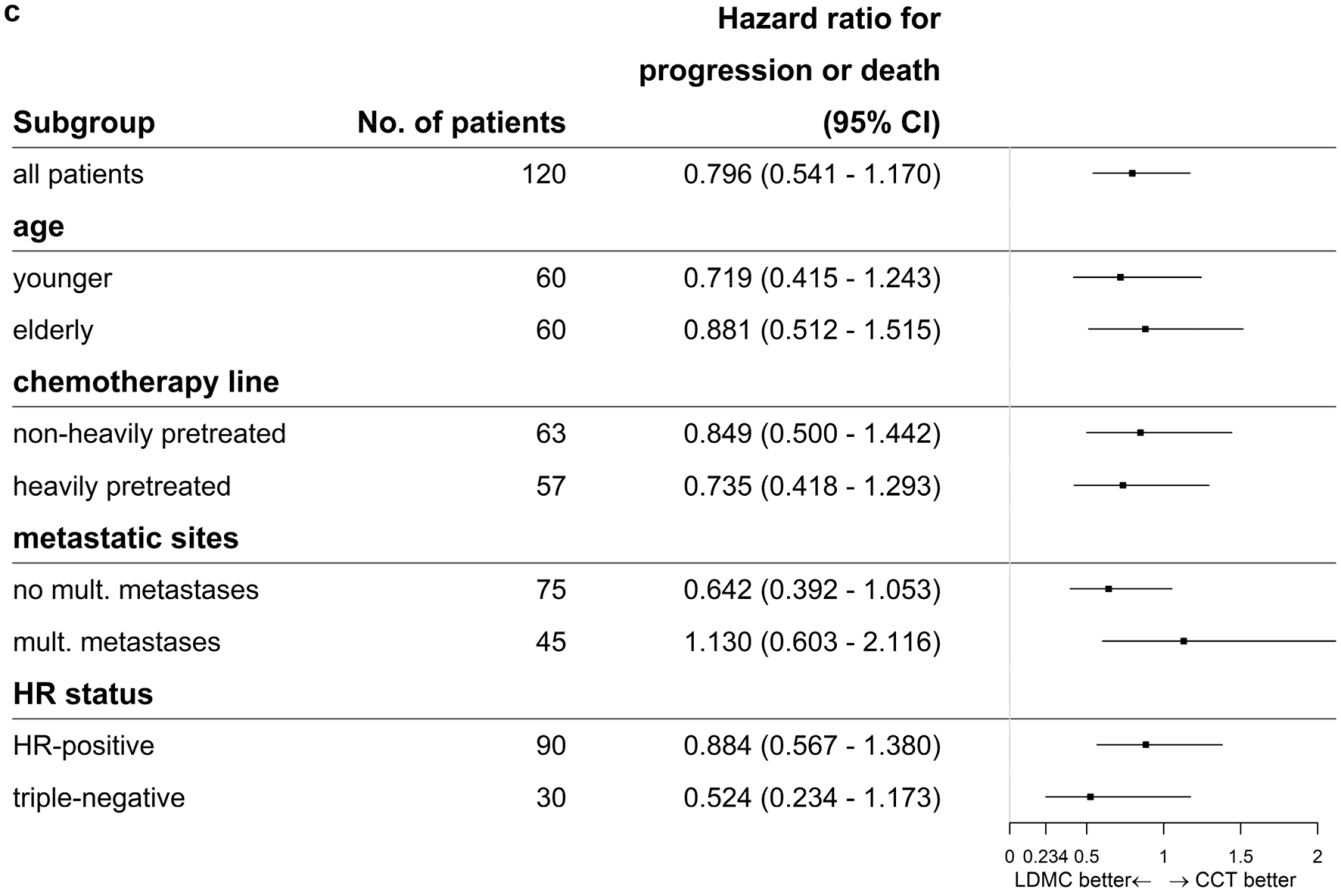
**a** Disease control rate in subgroups. **b** Median progression-free survival in subgroups. **c** Hazard ratio for progression or death in subgroups

In the subgroup analyses, 40.0% younger LDMC patients and 25.0% younger CCT patients showed DCR (*p* = 0.249) (Fig. 2a). 20.0% elderly patients achieved DCR in both treatment groups (*p* = 1.000). Among non-heavily pretreated patients, DCR was 33.3% in the LDMC and 26.2% in the CCT group (*p* = 0.568). In the heavily pretreated group, 26.3% vs. 18.4% patients showed DCR (*p* = 0.509). DCR was achieved in 36.0% LDMC patients and in 18.0% CCT patients (*p* = 0.096) without multiple metastases and in 20.0% vs. 30.0% with multiple metastases (*p* = 0.722). 30.0% vs. 28.3% HR-positive patients (*p* = 1.000) and 30.0% vs. 5.0% triple-negative patients achieved DCR (*p* = 0.095), respectively.

The median PFS in younger patients was 15.0 weeks in the LDMC group and 14.0 weeks in the CCT group (*p* = 0.212) (Fig. 2b), HR for progression or death was 0.719; 95% CI 0.415–1.243; *p* = 0.237 (Fig. 2c). Elderly patients showed a median PFS of 12.0 weeks in both groups (*p* = 0.627) (Fig. 2b). The median PFS in non-heavily pretreated patients was 17.0 weeks vs. 15.0 weeks (*p* = 0.531) (Fig. 2b), HR for progression or death was 0.849; 95% CI 0.500–1.442; *p* = 0.544 (Fig. 2c). In the heavily pretreated subgroup, the median PFS was 12.0 weeks for both treatment groups (*p* = 0.235) (Fig. 2b). The median PFS in patients without multiple metastases was 16.0 weeks vs. 12.0 weeks (*p* = 0.064) (Fig. 2b), HR for progression or death was 0.642; 95% CI 0.392–1.053; *p* = 0.079 (Fig. 2c). In the cohort with multiple metastases, the median PFS was 12.0 weeks in both groups (*p* = 0.684) (Fig. 2b). Regarding receptor status, the median PFS was 12.0 weeks vs. 14.0 weeks in the HR-positive group (*p* = 0.570) and 12.0 weeks in both triple-negative groups (*p* = 0.081) (Fig. 2b).

## Discussion

In this retrospective case–control study 120 MBC patients were evaluated regarding the efficacy of the chemotherapy treatment. The primary endpoint DCR did not differ significantly between LDMC and CCT group (30.0% vs. 22.5%, *p* = 0.380). The impact of metronomic CTX/MTX in our cohort of HR-positive and HER2-negative MBC patients as measured by DCR after 24 weeks of treatment was in line with previous studies [11–13]. Gebbia et al. [6] observed a higher PR rate in the cohort of patients with the combination CTX/MTX as compared to that treated with CTX alone (20% vs. 14%, *p* = 0.45). The median PFS was 12.0 weeks in the LDMC as well as in the CCT group (*p* = 0.218). Furthermore, DoR (31.0 vs. 20.5 weeks, *p* = 0.383) and therapy response (37.5% vs. 30.0%, *p* = 0.417) failed to show any significant differences between LDMC and CCT group. Moreover, the rate of treatment response may also depend on patient characteristics like age, metastatic spread, HR status as well as previous treatment. In the subgroup of younger patients, DCR was documented in 40.0% patients in the LDMC group and in 25.0% patients in the CCT group (*p* = 0.249). According to current recommendations for treatment of MBC, LDMC is primarily intended for elderly and frail patients, who are not suitable for conventional dosis of chemotherapy [14–16]. However, we have shown that LDMC can also be a treatment option for younger patients. Based on previous data from phase II studies, LDMC regimens provide promising results in the first-line setting with a clinical benefit rate (CBR) of up to 78% and a median time to progression (TTP) of up to 22 months [17–19]. Among the non-heavily pretreated subgroup, 33.3% LDMC patients and 26.2% CCT patients showed DCR (*p* = 0.568). More importantly, it is well established that the duration of disease control decreases with the increasing number of chemotherapy lines [20]. In the subgroup without multiple metastases, LDMC patients showed DCR twice as often as in the control group (36.0% vs. 18.0%, *p* = 0.096) and the median PFS was 16.0 weeks vs. 12.0 weeks (*p* = 0.064) with a trend towards significance. In the HR-positive group, we found no differences in DCR between the two groups. However, among triple-negative patients, 30.0% patients with LDMC compared to 5.0% patients with CCT showed DCR (*p* = 0.095) resulting in a borderline significance in favor of LDMC. A beneficial effect of the metronomic combination of VRL and CAPE was also shown in the triple-negative subgroup (28 patients) in the VICTOR-2 study [21]. The DCR was 53.7% and the median PFS was 4.7 months. Furthermore, LDMC with CTX/MTX was well-tolerable with almost only grade 1–2 toxicities. The most frequent adverse events were leukopenia (1–49%), nausea/vomiting (3–39%) and gastric pain (6–7%). Elevated values of transaminases, observed in up to 60% patients (10% grade 3–4), were mostly attributable to concomitant hepatic metastases or recovered with reduction or transient interruption of MTX [10, 12, 22]. In order to reduce hepatic toxicity and simplify the drug administration we modified the MTX schedule (2.5 mg every other day instead of 2.5 mg twice a day on days 1 and 4 every week) and found no grade 3–4 hepatic toxicities [10].

By reference to current experience, endocrine-based therapy should be provided as the first choice for MBC with positive HR status except in the case of life-threating disease [23]. In the last decade, new options as cyclin-dependent-kinase (CDK) inhibitors and immune checkpoint inhibitors for the treatment of MBC were established [24–26]. Moreover, LDMC has gained increasing interest through its multi-targeted nature. In addition to direct cytotoxic effect, LDMC induces indirect effects on tumor cells by modulation of tumor microenvironment via inhibition of angiogenesis and stimulation of immune response [27–29]. Thus, while the anti-tumor response to LDMC may be delayed, the effect is more likely to be sustained, owing to the decreased selection of resistant tumor cell clones and the suppression of anti-tumour immunity with a decreased likelihood of disease relapse [30]. Oral administration of well-tolerable LDMC including improvement of QoL of patients and reduced healthcare costs as well as having benefits over intravenous administration such as prolonged plasma drug concentration or increased therapeutic window makes LDMC attractive in clinical practice [31, 32]. Apart from that there are still several aspects that need to be clarified, such as patient selection, the choice of cytotoxic drug used for treatment, its optimal dose and decision between single versus doublet agent administration [8, 33]. Nevertheless, based on previous studies, LDMC represents a therapy option for MBC patients without need for rapid response and can be recommended according to the Breast Committee of the German Gynecological Oncology Working Group in HR-positive, HER2-negative MBC patients after anthracycline and taxane pretreatment [34, 35]. In addition to HR-positive patients, we demonstrated a favorable effect also in the triple-negative subgroup. Considering that, the therapeutic goal in advanced disease is on the one hand to maintain the QoL and on the other hand to control the disease, LDMC is a valuable option. It may be administered particularly in asymptomatic patients with endocrine resistance and triple-negative disease to prolong PFS and delay the onset of the often more toxic CCT with MTD regimen. Furthermore, combination of LDMC with anti-angiogenic and immunomodulatory substances seems to be promising [36–40]. Further analyses are needed to gain detailed experience about the role of LDMC in the management of MBC, including QoL issues and combination therapies with e.g. immunomodulatory drugs.

To the best of our knowledge, the presented analyses are the first to compare and show a similar efficacy of LDMC and CCT in terms of age, previous chemotherapy and severity of metastatic lesions. However, the retrospective character limits the validity of the presented data. In particular, since the median age at first diagnosis MBC was 59 for both groups and the median age at start of therapy was 63 in LDMC and 61 in CCT group, it can be assumed that LDMC patients had a less aggressive disease and/or better response to prior therapies compared to CCT patients. Reliable information on the toxicity of the administered therapies is not presented. Moreover, the validity of our conclusions is impaired by the study design and should be regarded as hypothesis generating. Therefore, we try to overcome this limitation and prepare a prospective non-interventional study to gather further insights about LDMC in MBC regarding patient-reported outcome, QoL, safety and efficacy, named PROmetronomic.

In conclusion, in our retrospective case–control study we could demonstrate a similar efficacy of LDMC compared to CCT in the treatment of MBC. Our analyses support further efforts to investigate the LDMC in selected MBC patients.
